# Microplastics Originating from Polymer Blends: An Emerging Threat?

**DOI:** 10.1021/acs.est.1c00588

**Published:** 2021-03-18

**Authors:** Xin-Feng Wei, Fritjof Nilsson, Haiyan Yin, Mikael S. Hedenqvist

**Affiliations:** † Fibre and Polymer Technology, KTH Royal Institute of Technology, SE−100 44 Stockholm, Sweden; ‡ Division Bioeconomy and Health, RISE Research Institutes of Sweden, SE−114 86 Stockholm, Sweden

## Abstract

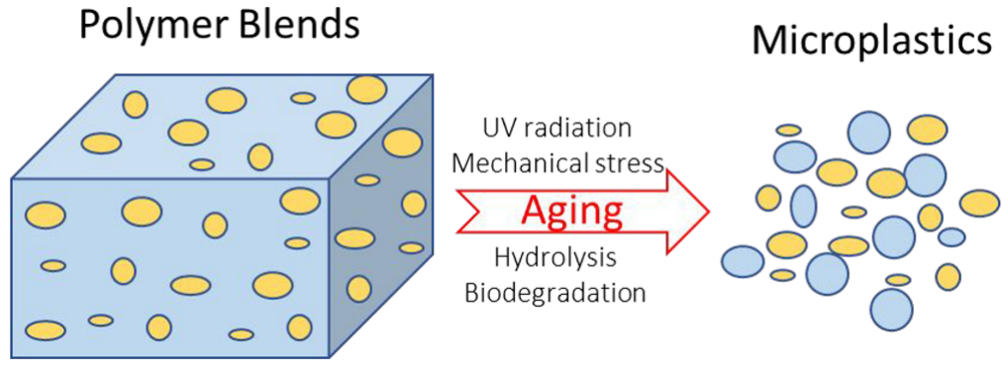

No one can have missed the growing global environmental problems with plastics ending up as microplastics in food, water, and soil, and the associated effects on nature, wildlife, and humans. A hitherto not specifically investigated source of microplastics is polymer blends. A 1 g polymer blend can contain millions to billions of micrometer-sized species of the dispersed phase and therefore aging-induced fragmentation of the polymer blends can lead to the release of an enormous amount of microplastics. Especially if the stability of the dispersed material is higher than that of the surrounding matrix, the risk of microplastic migration is notable, for instance, if the matrix material is biodegradable and the dispersed material is not. The release can also be much faster if the matrix polymer is biodegradable. The purpose of writing this feature article is to arise public and academic attention to the large microplastic risk from polymer blends during their development, production, use, and waste handling.

Polymer blending, mixing of at least two polymers or copolymers, is a very cost-effective, simple, and popular method to design/develop new materials for specific properties and applications.^1^ Being widely used in electronics, packaging, automotive, household appliances, etc., polymer blends (including plastic/plastic blends, plastic/rubber blends, and rubber/rubber blends) account for more than 20% of the total consumption of engineering polymers, and their future market is expected to continue to grow significantly.^2^ Besides, polymer blends are unintentionally and largely produced in the polymer recycling industry due to the difficulties of sorting various types of polymers. Most of the polymers are immiscible, and their blends show different morphologies such as the island-sea and cocontinuous structures depending on the ratio of polymer components, interfacial adhesion/tension, and processing parameters/protocols.^3,4^

The island-sea structure, consisting of a minor phase dispersed in a major continuous phase (as illustrated in Figure 1a), is the morphology for most polymer blends. For instance, it is the classic morphology of thermoplastic vulcanizates, in which cross-linked rubber particles are dispersed in a plastic matrix.^5^ The dispersed phase, in the form of spheres, cylinders, ribbons, and ellipsoids, has a size normally ranging from hundreds of nanometers to tens of micrometers.^3^ The morphology of the dispersed phase depends on several factors, including processing conditions and blend ratio.

**Figure 1:**
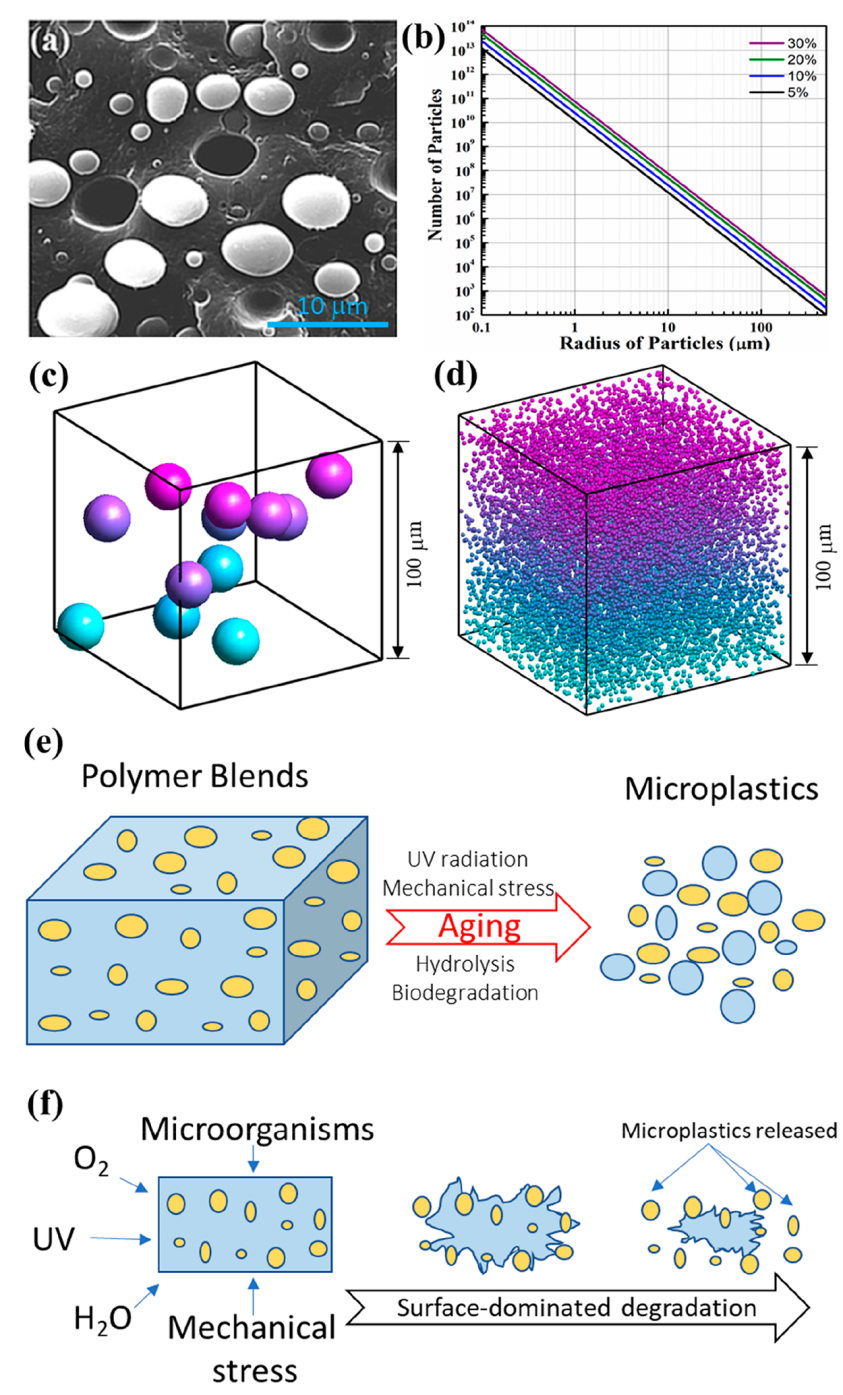
(a) The sea–island morphology in polymer blends illustrated by a scanning electron microscopy (SEM) image of a cryofractured surface of the common polypropylene/polycarbonate blend where the spheres (light gray) are the polycarbonate phase dispersed in a continuous polypropylene matrix (dark/black), (b) the number of polyethylene (PE) microplastic particles in 1 g of a polymer blend at different mass content of PE, plotted as a function of the average radius of particles. The 3D-view schematic of the particles with an average radius of (c) 1 and (d) 10 μm dispersed in a cubic polymer blend with a side length of 100 μm, and schematic of (e) the microplastics generated from polymer blends with a sea–island morphology during the aging and (f) the corresponding release process of microplastics from polymer blends under a surface-dominated degradation process. The SEM image in (a) is reproduced from ref (8) with permission from the PCCP Owner Societies. A density of 0.97 g/cm^3^ was used for the PE phase to calculate the number of microplastic particles in (b). A volume content of 5% was used for the dispersion phase of the polymer blend in c and d. Graphs c and d were generated with a straightforward Monte Carlo algorithm, which iteratively added impenetrable spheres at random positions in a periodic cubic domain such that no spheres became overlapping.

According to the definition of microplastics (size smaller than 5 mm),^6,7^ each individual dispersed particles in polymer blends should be regarded as a single piece of microplastic, even when they still reside in the continuous polymer matrix. The dispersed particles with a size smaller than 1 μm can be even regarded as a piece of nanoplastics. Due to their small size, the dispersed domains/particles are present in a very large number. The number (*n*) can be calculated from
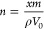
where *x* is the mass content of the dispersed polymer component in the polymer blend; *m* is the mass of the blend, ρ is the density of the dispersed polymer phase, and *V*_0_ is the average volume of the dispersed entities. Figure 1b shows an example of how the number of spherical polyethylene (PE) particles in 1 g of a polymer blend depends on the mass content of the PE phase and the average particle size/radius. The number of the PE microplastic particles increases linearly with decreasing particle size on a log–log scale since it is inversely proportional to the third power of its particle radius. Ca. 50 million spherical PE particles with a radius of 10 μm are contained in only 1 g of the polymer blend when the PE mass content is 20%, and this number rises to 50 billion for an average particle radius of 1 μm (Figure 1b). The abundant dispersed spherical particles in polymer blends are also illustrated in Figures 1c,d from a 3D view, where 12 and 12 000 particles are hidden in a polymer blend with a size of 100 × 100 × 100 μm^3^ for an average radius of 10 and 1 μm, respectively. The overall number of microplastic particles inside polymer blends is astronomical due to the large annual production of polymer blends (over 50 million tons^2^).

As long as the microplastic particles are still dispersed into the polymer matrix, they cause no troubles. The problem is that these large amounts of microplastics that are preserved inside the polymer blend can potentially be released into the environment during its production, when in service, when recycled, and at the waste stage, due to mechanical and environmental stresses, posing a high risk to the environment. The risk of microplastic migration is particularly high if the dispersed phase is significantly more stable than the surrounding polymer matrix. However, to our knowledge, no study has been conducted on these dispersed phases in polymer blends from the perspective of being a large source of microplastics.

## Microplastics Released from Polymer Blends during Degradation

Like single-component polymers, polymer blends undergo different types of degradation, including photodegradation, oxidation, erosion, and hydrolysis, depending on the type of polymer components and their environmental conditions, during the processing, storage, service, and as waste.^9^ The degradation causes polymer chain scission, and consequent fragmentation (such as surface powdering) of the plastic product, which process is well-known as the secondary source of microplastics. The adhesion between the dispersed and matrix phase is often poor, leading to that microplastic particles in the form of the dispersed phase are easily liberated from the polymer matrix when the latter fragments as a consequence of aging/degradation. The liberated particles of the dispersed phase accumulate in the environment together with the microplastic formed from the fragmentation of the polymer matrix (Figure 1e). The differences in degradation kinetics between the polymers in the matrix and dispersion phases play an important role in the release process of the dispersed microplastics. The risk of microplastic release is evident from the blend if the dispersed phase is significantly more stable than the surrounding polymer matrix. Depending on the materials and the environment, the dominating degradation mechanism can be biodegradation, thermal degradation, UV-light degradation, oxidative degradation, ozone-caused degradation, radiation-induced degradation, etc. Biodegradable polymers are often less stable than nonbiodegradable materials in representative environments, but not always. However, the type of degradation does not matter as long as the matrix degrades significantly faster than the dispersed phase.

Besides, polymer degradation is commonly a surface-dominated process where the surface suffers a higher degree of deterioration than the core part of the material.^10^ This will lead to a gradual release of dispersed microplastics from the degraded matrix surface of the polymer blend (Figure 1f). Moreover, because the dispersed phase is surrounded/protected by the polymer matrix, it may undergo degradation to a much lesser extent than the matrix polymer, especially in the case of surface-dominated degradation, and when being released, the less-degraded “dispersed” particles may likely be more persistent in the environment than the microplastics generated from the highly degraded polymer matrix.

## Microplastics in Biodegradable Polymer Blends

Environmental concerns over the pollution caused by traditional polymers have driven the fast development of biodegradable polymers. Different from traditional polymers, biodegradable polymers can break down into low molecular weight compounds such as water, methane, and carbon dioxide by microorganisms such as bacteria, fungi, and algae biodegradable polymers, which offers a possible solution to waste disposal problems associated with traditional plastics.^11,12^ Accordingly, various types of fully and partially biodegradable polymer blends emerge.^13^ For instance, nonbiodegradable rubbers such as acrylonitrile-butadiene rubber are commonly employed to toughen brittle biopolymers such as poly(lactide acid), one of the most commonly used biodegradable polymers.^14^ The dispersed particles can be released from the blends during biodegradation of the polymer matrix, for instance, in soil, water, and industrial composting sites, especially when these are not biodegradable or more slowly biodegradable than the matrix. Biodegradation is usually also a surface-dominated process,^14^ which can lead to a gradual release of dispersed microplastics from the polymer blend (Figure 1f). Biodegradation makes the matrix significantly less durable than in the case of nonbiodegradable polymer blends, which accelerates the release of the microplastics hidden inside the polymer blend, leading to immediate microplastic risk. Special concerns over the microplastic risk are needed during the development, production, use, and waste handling of biodegradable polymer blends. For instance, nonbiodegradable polymers should be avoided to be added into a biodegradable polymer matrix especially when the blends are designed for deposable products.
